# T Cell Involvement in Neuroinflammation After Traumatic Brain Injury: Implications for Therapeutic Intervention

**DOI:** 10.1111/cns.70580

**Published:** 2025-08-25

**Authors:** Mitchell D. Kilgore, Yuwen Xiu, Yinghua Jiang, Yingjie Wang, Mengxuan Shi, Di Zhou, Thin Sein, Sammy J. Vodovoz, Danni Wang, Aaron S. Dumont, Aimee Aysenne, Ning Liu, Xiaoying Wang

**Affiliations:** ^1^ Clinical Neuroscience Research Center, Departments of Neurosurgery and Neurology Tulane University School of Medicine New Orleans Louisiana USA; ^2^ Neuroscience Program, Tulane Brain Institute Tulane University New Orleans Louisiana USA; ^3^ Tulane University Translational Sciences Institute New Orleans Louisiana USA

**Keywords:** acute brain injury, neuroinflammation, T cells, T lymphocytes, traumatic brain injury

## Abstract

**Background:**

Traumatic brain injury (TBI) is a leading cause of death and disability across all age groups worldwide. After primary mechanical head injury, a cascade of molecular changes and immunological responses occur that are necessary for supporting tissue repair but also exacerbate the secondary loss of tissue caused by excessive neuroinflammation. To date, there are no targeted treatments that ameliorate the pathological neuroinflammation that is responsible for propagating secondary injury after TBI. Recent works have highlighted the adaptive immune system's response to TBI, with mounting evidence suggesting that T cells play a critical yet understudied role in propagating secondary injury while also potentially supporting reparative processes.

**Objectives:**

We critically review the current literature to discuss the diverse functionality of T cells in TBI including the temporospatial characteristics of their response, mechanisms of their activation, and their contributions to the overall neuroinflammatory profile. Consideration is given for additional pathological factors that may further alter these properties. We additionally summarize previous reports of therapeutic T cell modulation in this setting and identify approaches warranting additional investigation. Finally, we discuss major gaps in the existing literature and recommend future research perspectives.

**Conclusion:**

Evidence suggests several aspects of the T cell response to TBI may serve as beneficial therapeutic targets for limiting secondary injury. Additional translational investigations are warranted and may support the development of effective therapeutic strategies for treating patients post‐head trauma.

## Graphical abstract

Growing evidence suggests T cells play a vital role in shaping the neuroinflammatory milieu that develops following traumatic brain injury (TBI). Pharmacological approaches for therapeutically modulating the contributions made by T cells to the pathophysiology of TBI may identify promising interventional strategies for improving outcomes in TBI patients clinically.

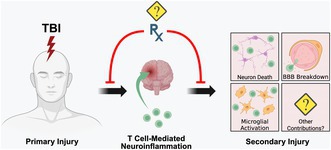

**Abbreviations**
APC: antigen presenting cell
BBB: blood brain barrier
CNS: central nervous system
DAMP: disease‐associated molecular pattern
IL: interleukin
MHC: major histocompatibility complex
MoA: mechanism of action
S1PR: sphingosine 1‐phosphate receptor
TBI: traumatic brain injury
TCR: T cell receptor
TLR: toll‐like receptor
Treg: regulatory T cell
γδ: gamma delta

## 1 Introduction

Traumatic brain injury (TBI) remains a leading cause of morbidity and mortality across all age groups worldwide [1] and epidemiological studies suggest that TBI prevalence will continue to rise as a result of a growing and aging population [2]. This poses a significant challenge for both afflicted patients, who are burdened with potentially life‐long deficits and an elevated risk of developing comorbid neurological sequelae later in life [3, 4, 5], and society as a whole, which is saddled with the substantial direct and indirect costs associated with TBI management [6]. Nevertheless, no targeted neuroprotective therapies exist to date for mitigating the grave consequences of TBI once head injury has been sustained.

Pathophysiologically, primary mechanical head injury triggers a multifaceted series of cellular and physiological changes that rapidly result in neuroinflammation [7]. Amongst these, central nervous system (CNS) antigens, danger‐associated molecular patterns (DAMPs), and cytokines released from damaged tissue quickly initiate inflammasome activation, which prompts activation and recruitment of CNS resident and peripheral immune cells [8]. Pro‐inflammatory microglial activation and neutrophil infiltration of the CNS occur within hours of primary injury, and peripheral monocytes, natural killer cells, and T cells respond shortly thereafter and continue to exacerbate neuroinflammation [8]. While necessary for clearing damaged tissue and initiating reparative processes, this inflammation also induces secondary brain injury, wherein otherwise viable tissue is harmed [9]. Thus, the detrimental components of neuroinflammation that develop following TBI serve as potential therapeutic targets for intervention.

Unlike many other brain‐infiltrating peripheral immune cells involved in the pathophysiological response to TBI, T cells are known to remain in the brain parenchyma for weeks to months after primary injury [10, 11, 12]. Importantly, T cells are functionally diverse and dynamic, serving both pro‐ and anti‐inflammatory roles in immunological diseases [13]. Evidence from other acute brain injuries has strongly implicated several major T cell populations in aggravating neuroinflammation and contributing to worsened outcomes, including CD8+ [14] and CD4+ [15] T cells. Likewise, in TBI, early evidence has demonstrated that both CD8+ and CD4+ T cells aggravate different aspects of the neuroinflammatory response including microglial activation, blood brain barrier (BBB) permeability, and neuron loss [11, 14, 16], though some subsets including regulatory T cells (Tregs) can have potent anti‐inflammatory functions [17]. Beyond this, however, many critical aspects of the T cell response are poorly understood, and it remains largely unstudied if targeted, T cell‐specific modulation is a viable clinical strategy for reducing secondary injury in TBI.

In this review, we comprehensively discuss the current state of evidence surrounding the role of T cells in TBI. We emphasize the temporospatial characteristics of this response, the mechanisms of T cell activation and recruitment to the CNS post‐TBI, and the ways in which unique T cell subsets exert their immunological functions. We additionally discuss biological and pathological factors that may influence aspects of this process and summarize previous therapeutic strategies that have been shown to alter T cell activity in this setting. We use this framework to identify key gaps in the current literature regarding the role T cells play in the pathophysiology of TBI and to propose clear research priorities for future investigations. These recommendations aim to advance our understanding of this unique adaptive cell population post‐trauma and support the development of effective therapeutic strategies for treating head injuries in the clinic.

## 2 The T Cell Response to TBI

### 2.1 Mechanisms of T Cell Activation

**FIGURE 1:**
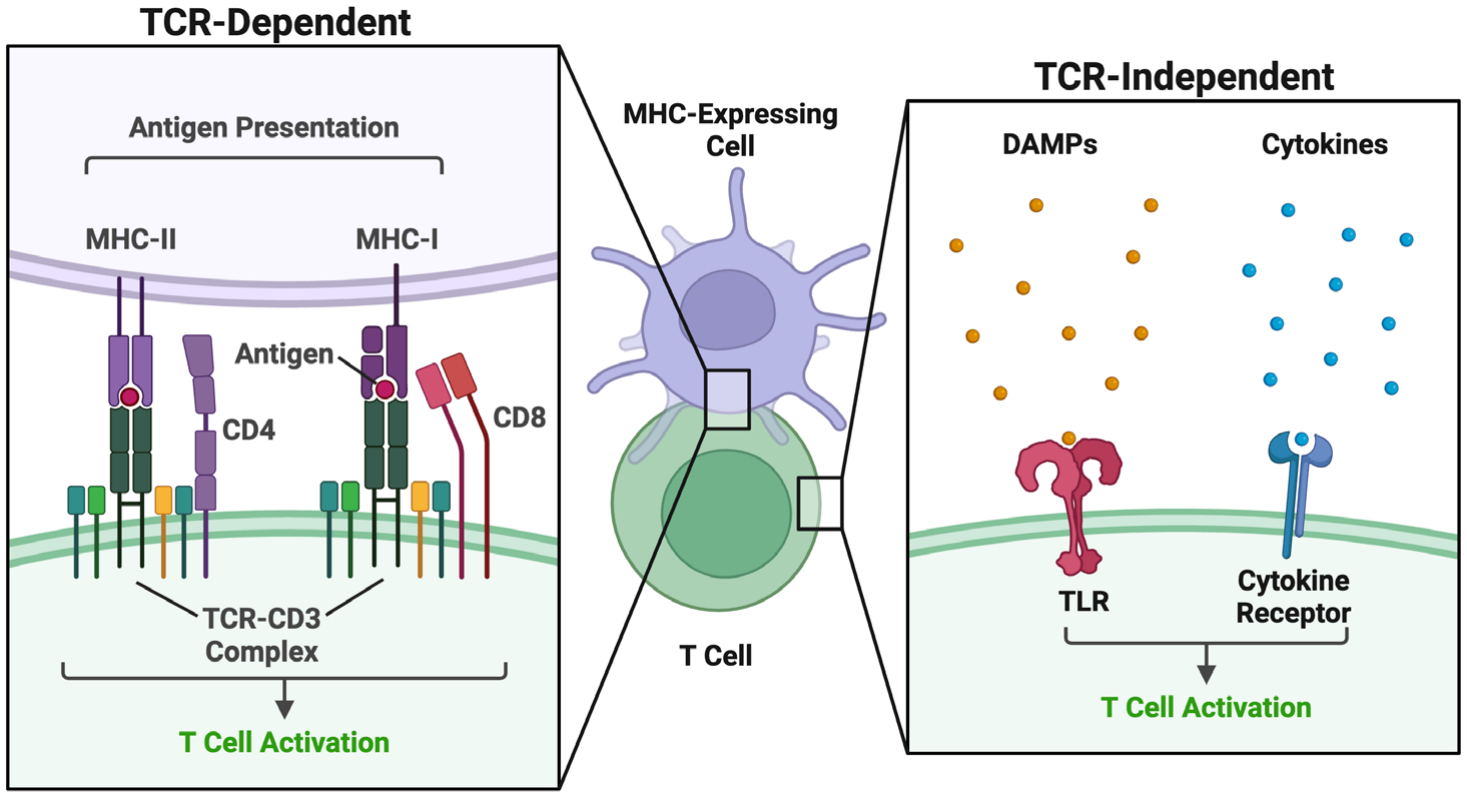
TCR‐dependent versus independent mechanisms of T cell activation. T cells are classically activated via TCR‐dependent mechanisms (*left*). Protein antigen is processed by MHC‐expressing antigen presenting cells and subsequently presented extracellularly on either MHC‐I or MHC‐II. The interaction of antigen‐loaded MHC‐I or II with the TCR‐CD3 complex on the surface of T cells and the co‐stimulatory signals CD8 or CD4, respectively, induces intracellular signaling cascades that ultimately promote cellular activation. T cells may also be activated in a TCR‐independent manner (*right*). Ligand cytokines and DAMPs released by other cells into the intracellular space bind to cytokine receptors or TLRs, respectively. This induces intracellular signaling to promote T cell activation distinct from the pathway initiated by the TCR complex. DAMPs, damage‐associated molecular patterns; MHC, major histocompatibility complex; TCR, T cell receptor; TLR, toll‐like receptor.

T cells are a unique population of adaptive immune cells that are derived from lymphoid progenitor cells and mature into specific functional subsets in the thymus during development before migrating to peripheral storage compartments [18]. In these locations, naïve T cells (e.g., mature T cells that have not previously become activated) remain functionally dormant and surveil the microenvironment in anticipation of immunological cues that induce their activation through several mechanisms. Classically, this activation has been shown to depend on direct interaction between the T cell receptor (TCR) complex on the T cell surface and cognate peptide antigens loaded in either major histocompatibility complex (MHC)‐I or MHC‐II of other cells [19, 20]. Alongside the TCR complex, the co‐receptor CD8 accommodates interaction with MHC‐I, which is expressed on all nucleated cells in mammals, whereas CD4+ T cells interact with MHC‐II, which is exclusively expressed on antigen presenting cells (APCs) [19, 20, 21]. These distinct T cell lineages respond to separate immunogenic cues, with CD8+ T cells primarily responding directly to cells presenting abnormal proteins of endogenous intracellular origin and CD4+ T cells responding to exogenous peptides that have been scavenged from the extracellular compartment and subsequently presented by APCs [20, 21]. However, after sufficient TCR stimulation and co‐stimulation have been achieved in both subtypes (Figure 1), the TCR complex initiates several intracellular signaling cascades that further activate distal transcription factors and other pathways that ultimately direct T cell expansion and phenotypic differentiation depending on the microenvironment's current cytokine signature [22, 23]. This adaptive process is unique in that, unlike in innate immune cell activation, it is highly specific to individual antigenic peptide sequences in a clonal fashion. This pathway, which has recently been shown to play a critical role in potentiating neuroinflammation following intracerebral hemorrhage [24, 25], may be particularly relevant in TBI. In this setting, primary injury instantly mechanically damages neurons and other cells within the CNS when the brain impacts the cranium and rotational forces from the impact are exerted within the parenchyma [26]. This leads to the immediate release of large quantities of potentially immunogenic proteins [27, 28], which, in turn, may amplify the magnitude of TCR‐dependent signaling compared to what is seen following cerebral ischemia, hemorrhage, or other isolated neuroinflammatory diseases. Hence, additional investigations into the role of TCR signaling and the therapeutic potential of its modulation in TBI are warranted.

In addition to TCR‐dependent activation, recent advances have highlighted the phenomenon of T cell activation occurring independent of TCR complex stimulation. DAMPs, endogenous stress signals released from dead and dying cells, enter the extracellular space and bind with several classes of toll‐like receptors (TLRs) on both innate and adaptive immune cells [29, 30]. DAMPs consist of cellular and extracellular components that are not exposed to surveilling immune cells under homeostatic conditions, including self‐DNA/RNA and structural proteins and peptides [29]. Additionally, cytokines released from other immune cells can directly stimulate cytokine receptors on T cells in a similar manner [31]. Upon successful interaction between DAMP/TLR or cytokine/receptor (Figure 1), numerous intracellular signaling pathways are activated that then promote upregulated transcription of numerous genes, including cytokines and interferons that cause both self‐activation and facilitate activation of neighboring immune cells [29]. As a conserved immunogenic pathway, this stimulation occurs in the absence of T cell interaction with a unique cognate antigen, as is seen through classical TCR‐dependent signaling.

### 2.2 T Cell Recruitment to the Injury Site

**FIGURE 2:**
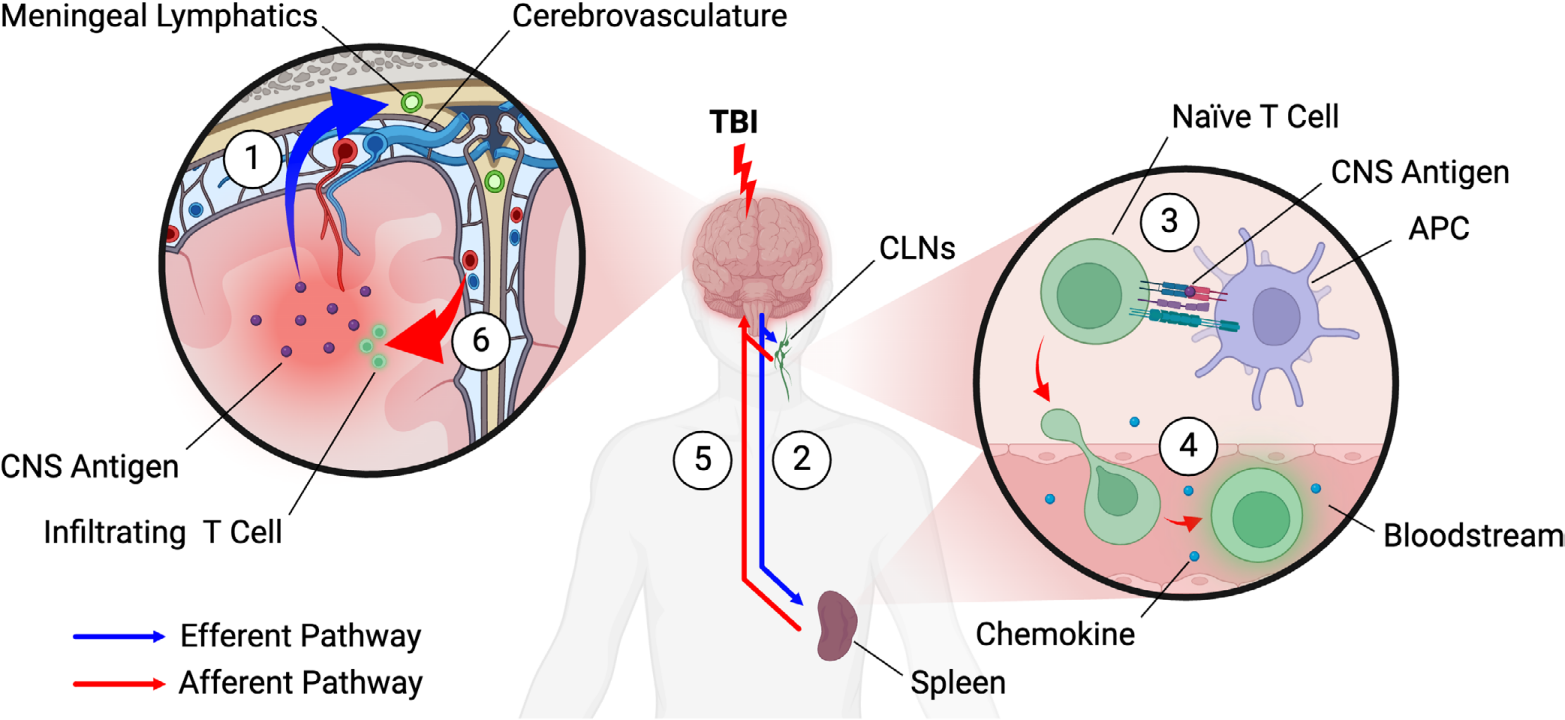
Potential mechanism of TCR‐dependent peripheral T cell recruitment after TBI. After primary mechanical injury, CNS antigens released from the parenchyma enter the meningeal lymphatics and bloodstream (1) where they are trafficked to secondary lymphoid organs like the CLNs and spleen (2). In these sites, APCs process and present the CNS antigen on MHC complexes to directly stimulate naïve T cells via the TCR (3). Chemokines concurrently released from the site of brain injury induce activated T cells to migrate into the bloodstream (4), where they home along the increasing chemokine gradient toward the brain (5) and ultimately infiltrate the parenchyma (6) to reach the site of injury. APCs, antigen presenting cells; CNS, central nervous system; CLNs, cervical lymph nodes; MHC, major histocompatibility complex; TCR, T cell receptor.

T cell recruitment to the injury site is complex and is an area warranting further scrutiny. It is known that brain resident T cells occupy a vital immunologic role in surveilling for and rapidly responding to brain injury [32, 33]. Nevertheless, it is becoming increasingly clear that peripheral T cell stores also play a major role in this response and are actively recruited into the CNS after acute injury [34, 35]. It has been shown that cytokines and DAMPs are rapidly released from damaged neurons and glia after acute brain injury, and these can interact with immune cells locally but may also travel to the periphery via the meningeal lymphatic system and bloodstream [36, 37, 38]. Once there, DAMPs and cytokines are likely capable of directly stimulating surveilling T cells within the circulation to induce their recruitment to the injury site via TCR‐independent mechanisms. However, because detectable quantities of numerous CNS‐derived antigens are also released into the blood and CSF after TBI [27, 28], it can be hypothesized that naïve T cells in peripheral lymphoid organs like the cervical lymph nodes and spleen may encounter immunogenic CNS antigen after it has been trafficked to the periphery and presented by APCs and subsequently undergo TCR‐dependent activation (Figure 2). Thereafter, activation of these naïve T cells would cause their differentiation, clonal expansion, and egress from the lymphoid tissues into the bloodstream, where they would then home towards the CNS via chemotactic signaling and ultimately infiltrate the parenchyma via the choroid plexus or neurovascular unit [39, 40, 41, 42]. Importantly, it has been shown that disrupting individual steps of this signaling pathway can directly interfere with effective recruitment of T cells to the brain after TBI. For instance, transgenic mice lacking meningeal lymphatics exhibit reduced CD4+ T cell brain infiltration post‐TBI and also lack T cell expansion in the cervical lymph nodes [43]. Cervical lymphadenectomy and cervical afferent lymphatic ligation can also both reduce post‐traumatic CD4+ T cell brain infiltration, reduce cerebral pro‐inflammatory cytokine expression, and improve neurobehavioral performance in mice, amongst other beneficial effects [38]. These suggest that a functionally intact brain to secondary lymphoid organ interface is necessary to effectively coordinate peripheral recruitment of T cells to the injury site, and this may indicate interventions targeting specific steps of this pathway have potential utility for altering the response of key T cell subset populations [38, 40, 43, 44]. Gamma‐delta (γδ) T cells likely respond from atypical lymphoid stores such as the calvarium and meninges in a similar manner [45], but the detailed mechanisms of their response in acute brain injury remain largely unstudied. Additional works are needed to elucidate the respective contributions of peripheral T cell stores versus brain and skull resident T cells to the overall T cell response to TBI. Furthermore, given the existence of multiple, distinct mechanisms of activation, additional investigations that evaluate the subset‐specific involvement of TCR‐dependent versus independent mechanisms focally at sites of neuroinflammation and in the peripheral compartments will be beneficial in TBI, as they may identify novel targets for intervention.

### 2.3 Temporospatial Characteristics of the T Cell Response to TBI

The recovery period following primary injury during TBI can be separated into acute, subacute, and chronic phases. These loosely correspond to the timeframes spanning the first 2–3 days, then out to around 1 month, and then beyond 1 month post‐injury, respectively; though it is important to note that this nomenclature varies across the preclinical and clinical literature. The temporospatial characteristics of T cell activation, differentiation, and recruitment to the CNS after TBI have been most extensively studied using rodent models, where injury parameters and genetic background can be highly controlled (Table 1). However, common trends in the temporospatial characteristics of the T cell response can be observed from the current literature, and studies have demonstrated that the response is dynamic and begins rapidly after TBI induction. In young mice, in which thymic involution has yet to occur, the thymic medullary area has been shown to increase significantly within the acute and early subacute phases [46]. In adult mice, T cells appear to undergo several rounds of proliferation in peripheral lymphoid tissues prior to mobilization during this timeframe as well [40]. Splenic stores of mature T cells then transiently decrease across the subacute phase following primary injury, which likely reflects their mobilization into the bloodstream and gradually decreased expansion [47]. Accordingly, levels of CD4+ and CD8+ T cells in the bloodstream may rise starkly within the acute and early subacute phases post‐injury and gradually return to pre‐injury levels [46, 49]; though this is not always the case, and levels of circulating T cells may remain relatively constant into the subacute phase [48]. One notable caveat lies in that, in severe TBI, peripheral immunosuppression and reduced T cell mobilization may actually be observed in the days that follow primary injury, which may be owed to the inhibition of T cell egress from secondary lymphoid tissues by elevated systemic catecholamine and cortisol levels [56, 57]. Within the brain parenchyma, elevations in the number of brain‐infiltrating T cells can be detected less than an hour after injury [51] and overall T cell infiltration peaks within the first 4 days of the subacute phase [11, 12, 47, 50, 51, 52, 53, 54, 55]. Thereafter, levels of both CD4+ and CD8+ T cells within the brain decrease substantially by around 1 week post‐injury; though several long‐term studies have indicated that levels of both subtypes remain elevated or even begin to rise bimodally well into the chronic phase after injury [10, 11, 12, 47, 53, 54]. Findings from these model studies agree with the limited published literature studying the temporal properties of the T cell response in human subjects [58, 59, 60, 61]. However, several critical components remain undescribed, including what the temporospatial characteristics of specific T cell subsets, including the CD4+ T cell subsets (e.g., Th1, Th2, Th17, Treg) and γδ T cells, are across this time frame and what role injury severity plays in directing the overall polarization of this process. The temporospatial patterns of T cell infiltration within the brain itself after TBI (e.g., T cell infiltration locally at the injury site versus other remote regions of the brain as secondary inflammation progresses) have not been adequately assessed, which may reveal associations between pathological features of TBI and functional outcomes, amongst other significant findings. The dichotomies between continued T cell infiltration versus intraparenchymal expansion and eventual egress versus apoptosis also warrant further scrutiny as they may alter the efficacy of targeting strategies at different time points post‐injury based on the intervention's mechanism of action (MoA).

**TABLE 1:** Summary of reported temporospatial observations of the T cell response to TBI in preclinical models.

Location	Subset	Subjects	Age	Sex	Model	Method	Observation	Ref.
Thymus	NS	Long Evans Rats	Juvenile (3 wk)	M	CHIMERA	IHC	The thymic medullary area expanded within 2 dpi	[46]
Spleen	NS	Long Evans Rats	Juvenile (3 wk)	M	CHIMERA	IHC	There was no difference in germinal center area or primary nodule area at 2 dpi	[46]
	CD3+	C57BL/6 Mice	Adult (8–10 wk)	M	CCI	FC	T cell numbers in the spleen fluctuated significantly over the first 7 dpi, remained decreased from 14 to 21 dpi, and returned to baseline at 28 dpi	[47]
CLNs	CD3+	C57BL/6 Mice	Adult (8–10 wk)	M	CCI	FC	There were significantly more T cells at 28 dpi compared to 1 dpi	[47]
PB	CD3+	Sprague Dawley Rats	Adult (12 wk)	M	CCI–R	FC	Total T cells decreased at 1 dpi, then continually increased to 14 dpi, then decreased below baseline at 28 and 42 dpi	[12]
	CD8+	Long Evans Rats	Juvenile (3 wk)	M	CHIMERA	FC	CD8+ T cells were only reduced at 2 h post‐TBI	[46]
		Sprague Dawley Rats	Adult (12 wk)	M	CCI–R	FC	Percentage of CD8+ T cells continuously increased from 7 to 42 dpi	[12]
		Sprague Dawley Rats	Adult (NS)	M	bTBI	FC	CD8+ T cells were only reduced at 2 h post‐TBI	[48]
	CD4+	Long Evans Rats	Juvenile (3 wk)	M	CHIMERA	FC	CD4+ T cells are elevated in the PB at 2 dpi	[46]
		Sprague Dawley Rats	Adult (12 wk)	M	CCI–R	FC	Percentage of CD4+ T cells continuously declined from 7 to 42 dpi	[12]
		Sprague Dawley Rats	Adult (NS)	M	bTBI	FC	CD4+ T cells were unchanged out to 3 dpi but reduced at 7 dpi	[48]
	Th2	C57BL/6 Mice	Adult (NS)	M	CCI	FC	Th2 polarization was unchanged at 1 and 21 dpi but decreased at 3 dpi	[49]
	Th17	C57BL/6 Mice	Adult (NS)	M	CCI	FC	Th17 polarization peaked at 1 dpi and remained elevated at 3 and 21 dpi	[49]
PB	Treg	C57BL/6 Mice	Adult (NS)	M	CCI	FC	Treg numbers were significantly reduced at 1, 3 and 21 dpi	[49]
		Sprague Dawley Rats	Adult (NS)	M	bTBI	FC	Tregs were only reduced at 3 dpi	[48]
Brain	CD3+	Long Evans Rats	Juvenile (3 wk)	M	CHIMERA	FC	There was no difference in total T cells at 2 dpi	[46]
		C57BL/6 Mice	Adult (8–10 wk)	M	CCI	FC	T cell numbers were transiently elevated across the first 7 dpi and again at 28 dpi, but were not elevated at 14 dpi	[47]
		C57BL/6 Mice	Adult (2 mo)	M	CCI	IHC	T cell numbers were elevated in both the ipsi‐ and contralateral hemispheres at 3 dpi	[50]
		Sprague Dawley Rats	Adult (12 wk)	M	CCI–R	FC	T cells were elevated at 1, 3, 7, 14, 28, and 42 dpi, with distinct peaks at 7 and 42 dpi	[12]
		C57BL/6 Mice	Adult (21 mo)	M	CCI	IHC	T cell numbers were elevated at 3 dpi in the ipsilateral but not the contralateral hemisphere	[50]
		Winstar Rats	Adult (NS)	M	WD–C	IHC	T cell numbers are transiently increased from 5 min to 1 dpi in numerous brain regions	[51]
		C57BL/6 Mice	Adult (NS)	M	cFPI	IHC	T cells were elevated in the perilesional cortex and subcortical white matter at 1 and 3 dpi but not at 7 dpi	[52]

	CD8+	C57BL/6 Mice	Juvenile (3 wk)	M	CCI	IHC	CD8+ T cells were elevated at 3 and 14 dpi but not 1, 7, or 35 dpi	[53]
		C57BL/6 Mice	Adult (9 wk)	M	CCI	qPCR	CD8+ T cells in the cortex peaked at 4 and 14 dpi but remained elevated above baseline from 7 to 35 dpi	[54]
		Sprague Dawley Rats	Adult (12 wk)	M	CCI–R	FC	Percentage of CD8+ T cells decreased to a low at 7 dpi before returning to baseline by 42 dpi	[12]
		C57BL/6 Mice	Adult (12 wk)	M	CCI	IHC	CD8+ T cells were elevated at 3 and 14 dpi but not 1, 7, or 35 dpi	[53]
		C57BL/6 Mice	Adult (7–13 wk)	M	CCI	FC	CD8+ T cells are elevated at 56 dpi	[10]
Sprague Dawley Rats		NS	M	WD	FC	CD8+ T cell infiltration peaked at 1 dpi but remained elevated through 3 dpi	[55]
Brain	CD4+	C57BL/6 Mice	Juvenile (3 wk)	M	CCI	IHC	CD4+ T cells peaked at 1 dpi, remained elevated at 3, 7, and 14 dpi, and returned to baseline at 35 dpi	[53]
		Sprague Dawley Rats	Adult (12 wk)	M	CCI–R	FC	Percentage of CD4+ T cells rose to a peak at 7 dpi before returning to baseline by 42 dpi	[12]
		C57BL/6 Mice	Adult (12 wk)	M	CCI	IHC	CD4+ T cells were elevated at 1 and 3 dpi but returned to baseline by 7 dpi	[53]
		C57BL/6 Mice	Adult (7–13 wk)	M	CCI	FC	CD4+ T cells were not elevated at 56 dpi	[10]
	Th1	C57BL/6 Mice	Adult (NS)	M	CCI	FC	Th1 polarization peaked at 1 dpi and were also elevated at 3 and 21 dpi	[49]
	Th2	C57BL/6 Mice	Adult (NS)	M	CCI	FC	Th2 polarization was unchanged at 1 and 7 dpi but was decreased at 3 and 21 dpi	[49]
	Th17	C57BL/6 Mice	Adult (7–13 wk)	M	CCI	FC	Th17 cells were elevated at 7 dpi	[10]
		C57BL/6 Mice	Adult (NS)	M	CCI	FC	Th17 polarization peaked at 1 dpi and remained elevated at 3 and 21 dpi	[49]
	Treg	C57BL/6 Mice	Adult (NS)	M	CCI	FC	Treg numbers were significantly reduced at 1, 3 and 21 dpi	[49]

Abbreviations: bTBI, blast‐induced closed‐head traumatic brain injury; –C, closed‐skull; CCI, controlled cortical impact; cFPI, central fluid percussive injury; CHIMERA, closed head injury model of engineered rotational acceleration; CLNs, cervical lymph nodes; FC, flow cytometry; h, hours; M, male; min, minutes; mo, months; NS, not specified; PB, peripheral blood; qPCR, quantitative polymerase chain reaction; −R, repetitive; WD, weight drop; wk, weeks.

## 3 Pathological Roles of T Cells in Secondary Brain Injury After TBI

**FIGURE 3:**
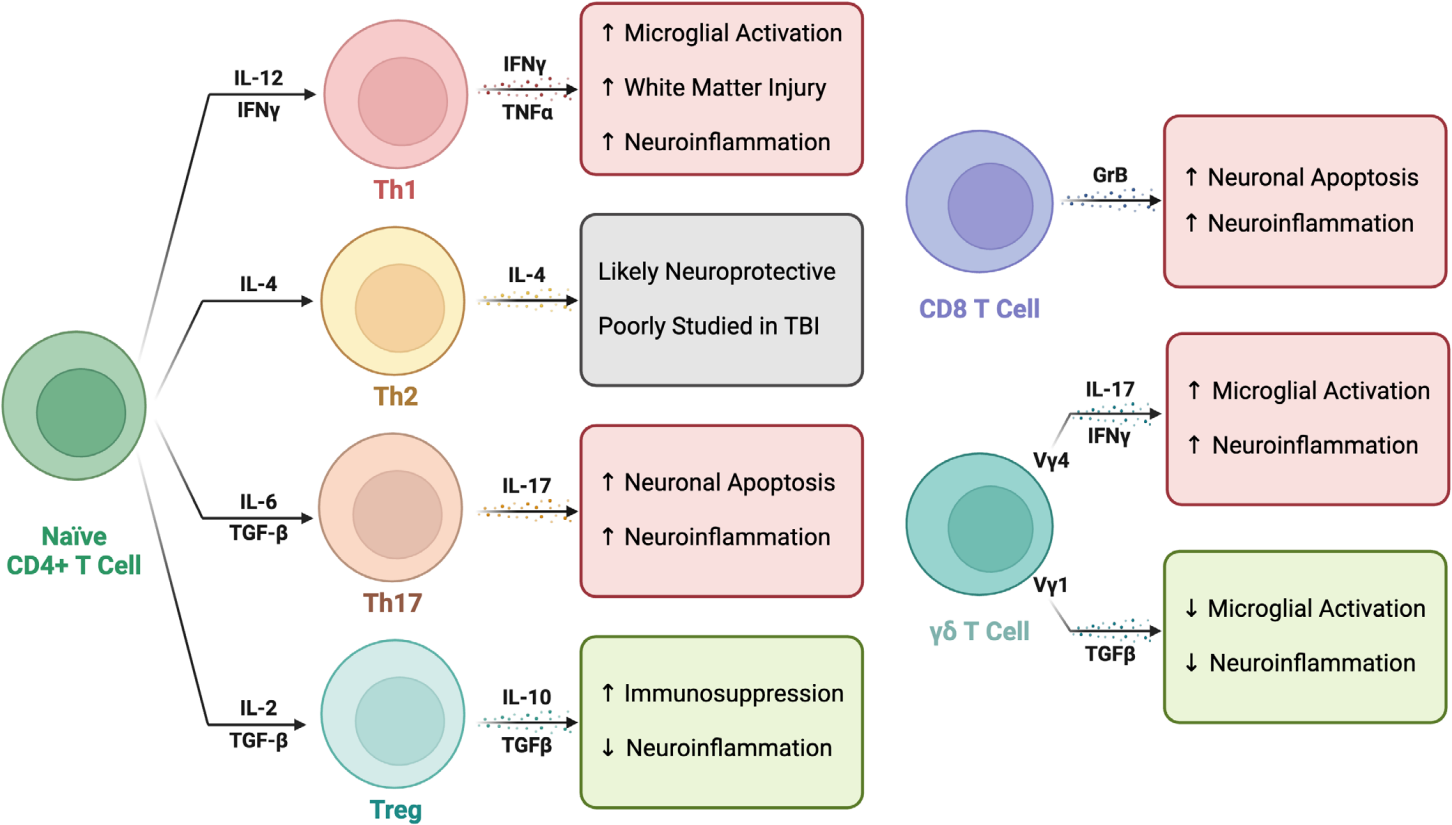
Contribution of T cell subsets to neuroinflammation in TBI. Upon successful activation, differentiation of naïve CD4+ T cells is directed by the chemokine profile of the microenvironment. Th1 and Th17 helper T cells have potent pro‐inflammatory functions in TBI, whereas Tregs have been shown to attenuate neuroinflammation. The role of Th2 helper T cells remains largely unstudied in TBI to date. CD8+ T cells exert pro‐inflammatory effects, and the functionality of γδ T cells depends on the variable portion of the γ chain of the T cell receptor.

Numerous mechanistic studies have sought to isolate and define the contributions of T cells to the overall post‐traumatic inflammatory milieu from those made by other innate and adaptive cell types. Observational studies have shown that the mere presence of CD3+ T cells, the broadest marker expressed on all T cell populations, within the brain parenchyma post‐TBI is associated with increased microgliosis [11] and corresponds closely with perilesional BBB dysfunction [11, 62]. However, the contributions made by this adaptive immune cell family are complex, given the diverse and dynamic functions of the many T cell subsets (Figure 3) [20]. Hence, there is a need to more finely consider the contributions made by each subset when considering the role and therapeutic potential in TBI.

### 3.1 CD4+ T Cells

Arguably the most phenotypically diverse of the T cell subsets, CD4+ T cells have been shown to make significant contributions to the neuroinflammatory profile that develops after TBI. Effector CD4+ T cells appear to directly aggravate secondary injury in the acute phase, likely due to augmented induction of neuronal apoptosis [38, 63]. As a whole, this suggests CD4+ T cells have a net pro‐inflammatory function in the acute phase; however, upon successful activation of a naïve CD4+ T cell in the presence of the proper microenvironmental cytokine signature, phenotypic differentiation is directed into several distinct subsets including Th1, Th2, Th17, and Treg, each with its own characteristics and functions [23]. Therefore, different CD4+ T cell subsets are likely to have diverse functions after TBI, with some directing pro‐inflammatory polarization and others attenuating this response. For instance, brain infiltrating Th1 cells have been shown to promote sustained microglial pro‐inflammatory activation in the acute phase of TBI via the secretion of IFNγ and TNFα, which is associated with increased demyelination and tissue loss compared to mice subjected to CXCL10 knockdown, a critical chemoattractant for this T cell population [44]. Th17 cells have similarly been shown to contribute to neuroinflammation in TBI, where levels of their effector cytokine interleukin (IL)‐17a are elevated within the first 6 h post‐injury and are associated with increased neuronal apoptosis and overall secondary injury severity [64]; though, surprisingly, one study found that genetic knockout of IL‐17 was not intrinsically neuroprotective [65]. In contrast, Tregs exert potent anti‐inflammatory effects in TBI through the production of IL‐10 and TGFβ, which enable them to directly attenuate the pro‐inflammatory functions of other cells including microglia and other T cells [66]. This function is augmented via the alarmin IL‐33, which has recently been highlighted as an early immune signal released from damaged neurons and glia in acute brain injury [66, 67]. Depletion of Tregs in TBI has detrimental consequences including enhanced brain infiltration of effector T cells, increased reactive astrogliosis, and greater BBB permeability, which contribute to worsened motor performance in the acute phase [68]. Conversely, transfer of umbilical cord blood‐derived Tregs in TBI models has been shown to dramatically reduce neuroinflammation by regulating numerous immune cell populations [69, 70, 71, 72]. Clinically, circulating Treg levels are positively correlated with improved outcomes in TBI patients [73] and severe TBI is associated with impaired Treg function, potentially exacerbating the response of other T cell populations [74]. Taken together, these findings show that the phenotypic differentiation of naïve CD4+ T cells directly influences the pro‐ versus anti‐inflammatory functions of the T cell response to acute brain injury and suggests strategies that aim to reduce effector CD4+ T cell differentiation while promoting Treg expansion may prove beneficial in TBI by augmenting the neuroprotective aspects of this response. Furthermore, while Th2 cells have been shown to be beneficial in other models of acute brain injury [75], to the best of our knowledge, no studies looking into Th2 cells in TBI exist to date, and this is an area in need of additional investigation in the future.

### 3.2 CD8+ T Cells

To date, there are a limited number of studies that have focused on the function of CD8+ T cells in the recovery period following TBI. While cross‐presentation of exogenous antigen on MHC‐I by APCs to accommodate recognition by CD8+ T cells is a known phenomenon [76], these cells primarily respond to abnormal proteins of intracellular origin, as seen in viral infection [21]. Hence, it remains unclear which immunogenic cues initiate their response in TBI and what mechanisms are responsible for their activation. Functionally, it is known that binding of pro‐inflammatory signals including IL‐15 released from activated astrocytes in response to primary injury can induce CD8+ T cells to secrete granzyme B into the extracellular space [10, 77]. This in turn activates caspase‐3 mediated apoptotic signaling cascades in other local cells including neurons [77]. Notably, this process in TBI is long lasting, as significantly increased levels of CD8+ T cells are detectable in the ipsilateral hemisphere as long as 8 weeks post‐injury in CCI mice [10]. Depletion or genetic deficiency of CD8+ T cells appears to be neuroprotective and is associated with improved neurobehavioral performance, reduced effector CD4+ T cell differentiation and brain infiltration, and reduced white matter injury at chronic timepoints suggesting CD8+ T cells may be a viable target for reducing chronic neuroinflammation after TBI [10]. However, while it has been shown that CD8+ T cells can undergo continued intraparenchymal proliferation after their initial infiltration and persist within the brain for extended periods [40], findings from a recent study suggest that their chronic presence is largely due to continued replenishment from the periphery [78]. This discrepancy is highly translationally relevant as it may predilect certain intervention strategies over others (e.g., targeting peripheral CD8+ T cell activity versus the activity of CD8+ T cells that are already present in the brain) for targeting the chronic neuroinflammation driven by this cell population. In light of this, additional studies that thoroughly investigate both the mechanisms and role of CD8+ T cells and their potential as a therapeutic target after TBI are warranted.

### 3.3 γδ T Cells

Possessing TCRs comprised of a γ and δ chain (as opposed to that of CD4+ and CD8+ T cells which are comprised of an α and β chain), γδ T cells are non‐conventional in that they are lowly abundant in the peripheral blood and instead are primarily tissue‐resident immune cells that home to peripheral tissues in lieu of traditional secondary lymphoid organs after development in the thymus [79, 80]. γδ T cells express TCRs of relatively low diversity to rapidly respond to distinct cognate antigens, causing them to be described as “innate‐like” and acting as a bridge between innate and antigen‐specific adaptive immune cells [81, 82]. Hence, this T cell population is uniquely poised to act as a first responder to injury within the anatomical compartment in which it resides, including the skull and CNS [79, 83]. However, despite evidence that suggests γδ T cells are important contributors to neuroinflammation in other acute brain injuries [84, 85], little is known about their role in TBI. Indeed, parenchymal γδ T cells have been shown to increase in number within the first 24 h after head injury [86]. Interestingly, TBI was associated with reduced expression of MHC‐II on γδ T cells, which was proposed to be indicative of reduced antigen processing capability and a respective decreased ability to couple innate and adaptive immune responses early after injury, potentially worsening secondary injury [86]. Using γδ knock‐out mice, another study found that γδ T cells worsened neurobehavioral performance in terms of motor function, anxiety‐like behaviors, and memory at chronic time points post‐TBI, though these findings were only significant in male mice [87]. This study also demonstrated that γδ knock‐out mice exhibited reduced acute brain infiltration of both CD4+ and CD8+ T cells, neutrophils, and monocytes, as well as reduced microglial activation at acute and chronic timepoints, supporting the hypothesis that γδ T cells are integral for acutely coordinating both innate and adaptive immune responses after injury. However, the neuroinflammatory functions of γδ T cells were shown to be dependent on the variable portion of the γ chain of the TCR, with Vγ4 γδ T cells potentiating neuroinflammation through the production of both IL‐17 and IFNγ and Vγ1 γδ T cells exerting anti‐inflammatory effects via the expression of TGFβ. Thus, additional works are needed to better understand the functional significance of these paradoxical functions and to further elucidate the dynamic response of this unique T cell subset in TBI.

## 4 T Cell Interplay With Other Biological and Pathological Factors After TBI

Unlike in models of TBI, which are highly controlled to reproducibly induce homogenous injuries across subjects [88], human TBI is extremely heterogenous [1]. Thus, differences in injury mechanism, location, and severity are known to confound the study of TBI in humans [89] and these factors undoubtedly confound the study of the T cell response clinically. Indeed, preclinical modeling has demonstrated that altering injury mechanism and severity directly influences the cellular and molecular changes that occur following primary injury [90]. However, in addition to primary injury heterogeneity, numerous other factors exist that may also influence the T cell response to TBI.

### 4.1 Age

This response has recently been shown to be age‐dependent, with the effectiveness of the response decreasing with age [50, 53, 91, 92, 93, 94]. Preclinical evidence has shown that the number of perilesional brain‐infiltrating T cells is higher in young animals compared to aged animals acutely [50], but that T cell infiltration and activation are significantly higher in aged animals at chronic time points [92]. Analysis of age‐dependent changes in the meningeal transcriptome has further shown that aging alone alters the meningeal compartment and primes the adaptive immune system for an exaggerated, pro‐inflammatory response after TBI [91]. These findings corroborate those from several clinical studies, which have shown that younger patients with TBI are more likely to develop autoreactive T cells against myelin basic protein compared to older patients, which is associated with better prognosis and may reflect a protective autoimmune function [94]. Moreover, older patients exhibit altered T cell activation patterns compared to younger patients, with more T cells differentiating into phenotypes with low cytotoxic activity [93]. These may partially explain why older TBI patients have higher morbidity and mortality rates than their younger counterparts [95]. However, further studies are needed to better understand the mechanisms that underlie this association and to determine if age may alter the efficacy of pharmacologically targeting subsets of T cells in TBI recovery, as has been observed in preclinical studies [78, 96].

### 4.2 Genetics

Host genetics are likely to be highly influential in shaping the T cell response. Allelic differences in the *Ciita* gene, the master regulator of MHC‐II expression [97], were shown to regulate MHC‐II expression in the injured cortex by controlling mRNA expression of the invariant chain in a rat TBI model [98]. In turn, the degree of MHC‐II expression strongly correlated with the magnitude of T cell infiltration of the brain post‐injury [98]. This is clinically relevant given the highly heterogenous genetic background of human subjects and may explain differences in the T cell response between otherwise similar patients with similar injuries. Studies assessing allelic differences in the *Ciita* gene and other genes that influence the T cell response in TBI are warranted, and bioinformatics analysis of large omics datasets may be helpful for identifying such targets of interest [99, 100, 101, 102].

### 4.3 Sexual Dimorphism

Immune cell composition and function vary between biological sexes and are dependent on complex contributions from both sex‐specific hormones and the differential expression of X chromosome‐linked immune‐related genes [103, 104]. In health, it has been shown that basal levels of T cell subsets in the circulation and secondary lymphoid structures differ between male and female subjects [105]. However, this immunological sexual dimorphism also has broad implications across numerous infectious and inflammatory diseases, including acute brain injury [106, 107, 108, 109]. To date, the influence of sex on outcomes after clinical TBI remains controversial; the majority of preclinical studies are modeled in male animals under the justification that TBI is more predominant in male patients [110]. Indeed, in the temporospatial studies reviewed (Table 1), 100% of the data was derived from male animals. Thus, immunological sexual dimorphism in TBI remains largely unexplored, and investigations using both male and female subjects aimed at understanding this phenomenon's potential influence on the T cell response in this setting are likely to provide valuable insights with relevance for therapeutics development.

### 4.4 Medical Comorbidities

Common metabolic and cardiovascular comorbidities are known to alter T cell homeostasis and function. For example, hypertensive patients show a significant loss of regulatory T cells accompanied by expansion of pro‐inflammatory Th17 cells at baseline [111]. Likewise, type 2 diabetic patients often have chronically elevated Th1 and Th17 effector T cells with concomitant loss of naïve and Treg populations [112]. In dyslipidemia, excess cholesterol can amplify TCR signaling and boost CD8+ T cell effector function, while atherogenic lipids can prime dendritic cells to secrete cytokines that drive differentiation of inflammatory helper T cell subsets [113]. These examples illustrate well‐established links between frequently encountered comorbidities seen in TBI patients and altered T cell function. As a result, these and other comorbidities likely further influence the T cell response to TBI and should be areas of further inquiry.

## 5 Development of T Cell Targeting Strategies Post–TBI

Numerous studies have investigated the therapeutic potential of pharmacologically targeting secondary injury in TBI, and several of these approaches have been shown to alter the T cell response through both direct and indirect modulation. In general, these approaches aim to either reduce effector T cell functioning or bolster the response of Tregs to reduce neuroinflammation. However, the majority of these studies use treatments with MoAs that are not specific for T cells [44, 55, 114, 115, 116, 117, 118, 119, 120, 121, 122, 123, 124, 125]. For instance, progesterone administration after TBI was recently shown to increase splenic Treg numbers and improve performance on the Morris water maze [114]. Other nonspecific medications including atorvastatin, propofol, dexmedetomidine, doxycycline, and esketamine have also been shown to augment the T cell response to TBI, amongst other effects [115, 116, 121, 122, 125, 126]. Because the MoAs of these medications are not specific to T cells, however, the terminal phenotype of treated subjects undoubtedly receives multifaceted contributions from numerous impacted cell types, and this makes it difficult to isolate potential contributions achieved by altering the T cell response.

Several approaches using interventions with MoAs that target leukocyte activity with higher specificity have been employed in preclinical models of TBI (Table 2). The anti‐α4‐integrin antibody natalizumab, which blocks immune cell diapedesis through the BBB, was recently shown to improve survival and neurobehavioral performance in aged mice at two months post‐injury and, interestingly, augmented the Th2 response in the plasma while reducing the number of CD8+ T cells in the brain at this time point [96]. Further experiments determined that the reduction of CD8+ T cells was a result of their reduced chronic infiltration as opposed to increased resident T cell proliferation, which is consistent with the intervention's peripheral MoA [78]. A similar approach using sphingosine 1‐phosphate receptor (S1PR) antagonists including fingolimod and siponimod, which inhibit lymphocyte egress from lymphoid organs and thereby induce immunosuppression, has also been employed in TBI [130, 131, 132, 133]. These studies found that S1PR antagonism improved a wide array of post‐injury outcome measures including lesion volume, neurobehavioral performance, BBB integrity, cytokine profile, cerebral edema, and neuronal apoptosis, amongst others [130, 131, 133]. This conferred neuroprotection was further shown to occur at least in part through S1PR antagonists' ability to modulate the PI3K/AKT pathway, which regulates several important physiological processes surrounding cell survival [130, 134]. However, an additional study did not identify any neuroprotective benefits of fingolimod after focal cortical cryolesion or weight drop models, which assess for focal and diffuse injury respectively [132]. It is possible that differences in TBI modeling methods confounded these findings, as two of the three studies showing S1PR antagonism was beneficial used the controlled cortical impact model of TBI, which delivers a moderate to severe mechanical injury directly to the exposed cortical surface [131, 133], whereas the focal cortical cryolesion and weight drop models were both performed closed‐skull in the study that found no effect [132]. Thus, differences in injury severity could explain these conflicting findings and studies that investigated this further are likely to be informative.

**TABLE 2:** Summary of reported experimental strategies targeting the T cell response in TBI.

Category	Intervention	Subjects	Observed effects on T cells	Effects on outcomes after TBI	Ref.
Astrocyte‐secreted IL^a^	IL‐2	CCI Mice	↑ brain Treg percentage	↓ microglial activation, ↓ lesion volume, ↑ neurobehavioral performance	[127]
Calcineurin inhibitor	Cyclosporin A	ACI Mice	Np	↓ lesion volume, ↓ neuronal apoptosis	[63]
Exogenous IL	IL‐2C	CCI Mice	↑ brain‐infiltrating Tregs, ↑ peripheral/splenic Tregs, ∅ peripheral blood/splenic CD4+ T cells, ∅ peripheral blood/splenic CD8+ T cells	↓ brain‐infiltrating neutrophils, ↑ neurobehavioral performance, ↑ BBB integrity, ↓ cerebral edema, ↓ pro‐inflammatory cytokines, ↑ anti‐inflammatory cytokines	[128]
Exogenous IL	IL‐33	CCI Mice	↑ brain‐infiltrating Tregs	↓ lesion volume, ↑ neurobehavioral performance, ↑ anti‐inflammatory cytokines	[66]
MHC‐II Inhibitor	CAP	FPI Mice	↓ splenic CD4+ T cells, ↓ splenic CD8+ T cells, ↓ splenic Tregs, ↓ γδ T cells	↓ neurodegeneration, ↓ lesion volume, ↓ pro‐inflammatory cytokines	[86]
Monoclonal Antibody	Anti‐α4‐Integrin (Natalizumab)	CCI Mice	↑ chronic peripheral blood Th2 cells, ↓ chronic brain‐infiltrating CD8+ T cells	↑ survival, ↑ neurobehavioral performance	[96]
Monoclonal Antibody	Anti‐α4‐Integrin (Natalizumab)	CCI Mice	↓ chronic brain‐infiltrating CD8+ T cells, ↓ total brain T cells	↑ survival, ↑ neurobehavioral performance	[78]
Monoclonal Antibody	Intranasal Anti‐CD3	CCI Mice	↑ meninges and brain‐infiltrating Tregs	↑ neurobehavioral performance, ↓ pro‐inflammatory microglial activation	[129]
S1PR Antagonist	Fingolimod (FTY720)	WD Mice	Np	↑ neurobehavioral performance, ↓ cerebral edema, ↓ lesion volume, ↓ neuronal apoptosis	[130]
S1PR Antagonist	Fingolimod (FTY720)	CCI Mice	↓ brain‐infiltrating T cells, ↑ Treg percentage	↑ BBB integrity, ↑ neurobehavioral performance, ↓ microglial activation, ↓ brain cytokines	[131]
S1PR Antagonist	Fingolimod (FTY720)	FCC Mice & WD Mice	↓ peripheral blood T cells	∅ BBB integrity, ∅ neurobehavioral performance, ∅ lesion volume, ∅ neuronal apoptosis	[132]
S1PR Antagonist	Siponimod	CCI Mice	↓ brain‐infiltrating CD4+ T cells, ↓ brain‐infiltrating CD8+ T cells	↓ microglial activation, ↓ astrocyte activation, ↑ BBB integrity, ↓ lesion volume, ↓ pro‐inflammatory cytokines	[133]
S1PR Antagonist	TASP0277308	CCI Mice	↓ brain‐infiltrating CD4+ T cells, ↓ brain‐infiltrating CD8+ T cells	↓ microglial activation, ↓ astrocyte activation, ↑ BBB integrity, ↓ lesion volume, ↓ pro‐inflammatory cytokines	[133]

Abbreviations: ∅, no difference; ↑, increased; ↓, decreased; ACI, aseptic cerebral injury; BBB, blood brain barrier; CAP, competitive antagonist peptide; CCI, controlled cortical impact; FCC, focal cortical cryolesion; FPI, fluid percussion injury; IL, interleukin; IL‐2C, IL‐2/Anti‐IL‐2 complex; MHC, major histocompatibility complex; Np, not provided; S1PR, sphingosine 1‐phosphate receptor; Treg, regulatory CD4+ T cell; WD, weight drop; γδ, gamma delta.

^a^ Via CNS gene delivery platform that causes locally activated astrocytes to secrete IL‐2.

Strategies aimed to specifically target T cells in TBI are less common. The calcineurin inhibitor cyclosporin A has been shown to induce T cell inhibition that is associated with reduced lesion volumes and neuronal apoptosis compared to untreated animals [63]. However, while one clinical study found that cyclosporin A treatment after TBI did not induce further peripheral immunosuppression in severe TBI patients [59], the immunosuppression caused by severe TBI already places these patients at increased risk of severe hospital‐acquired infection [135] and the potential reduction in the body's ability to respond to foreign organisms owed to cyclosporin A treatment limits its practical utility in the clinic. Another approach for T cell inhibition utilized a novel peptide‐based MHC‐II inhibitor that specifically blocks APCs' ability to load processed antigen onto MHC‐II for presentation to surveilling CD4+ T cells, thereby inhibiting their activation [86]. This strategy reduced splenic expansion of several T cell subsets and ultimately reduced neurodegeneration, lesion volumes, and pro‐inflammatory cytokine production within the brain. However, because this approach only mitigates TCR‐dependent activation of CD4+ T cells, it does not address the potentially damaging effects of CD8+ or γδ T cells, which is a limitation of the approach. Alternatively, several groups have focused on exploiting pathways involved in Treg activation and infiltration to augment their anti‐inflammatory properties. For example, treatment with either IL‐33 or IL‐2 has both been shown to be beneficial in TBI as they bolster the function of Tregs to reduce the pro‐inflammatory activation of other immune cells [66, 127, 128]. Translationally, peripheral injection of these cytokines is limited by both their relatively short half‐life in the bloodstream and their potential for causing excess immunosuppression in TBI patients. To overcome this, a novel gene delivery platform that specifically enhances astrocytic expression of IL‐2 only at sites of astrogliosis was developed, which bypasses the periphery and acts primarily on brain‐resident Tregs [127]. When applied in TBI, this was shown to limit microglial activation, reduce lesion volumes, and improve neurobehavioral performance compared to mice receiving gene delivery with the sequence encoding IL‐2 omitted. Another approach utilizing intranasally administered monoclonal anti‐CD3 antibody, which is known to induce differentiation of Tregs [136], was recently shown to increase the number of Tregs that migrate into the injured brain after TBI [129]. IL‐10 secreted from these cells decreased pro‐inflammatory microglial activation and enhanced microglial phagocytic capacity, leading to improved cognitive and motor recovery compared to untreated mice. Collectively, these studies highlight the therapeutic utility of targeting specific components of the T cell response to TBI and warrant further exploration.

## 6 Limitations of the Current Literature

In addition to the previously discussed limitations inherent in preclinical TBI research introduced by differences in preclinical model selection and cohort selection, several additional important factors should be considered when critically appraising the current literature. It is well established that poor experimental rigor and subsequent reproducibility hampers translational neuroscience research [137]. While certainly not universal (e.g., [87, 127, 129]), many studies reviewed here and within the translational sciences as a whole under‐describe or omit key scientific details that limit the reproducibility of their experimental results and the generalizability of their findings. For instance, the majority of studies included in this review did not provide scientific rationale for sample size selection as a component of the study's methods. Failing to provide such information increases concern for selective interference in data reporting and is avoidable with transparent reporting practices [138]. Moving forward, we emphasize that it is critical for authors to include descriptions of key methodological and interpretation components of their study, including descriptions of statistical analyses performed, rationale for sample size and cohort sex selection, experimenter blinding protocols, subject exclusion criteria, and their own critical appraisal of their study's limitations, amongst others. Doing so will help limit the overinterpretation of future study findings pertaining to the role of T cells in TBI to best facilitate the discovery of viable therapeutics.

## 7 Future Research Perspectives

Despite the growing body of evidence that has implicated T cells as key players in mediating the neuroinflammatory profile that contributes to secondary brain injury following TBI and demonstrated that targeting T cells in this setting may be a viable therapeutic strategy for limiting neuroinflammation to improve outcomes, several critical areas of focus remain unexplored.

Evidence surrounding the mechanisms of the T cell response to TBI and the diverse ways they interact with other immune and non‐immune cell populations within the brain and meninges remains relatively superficial. To overcome this, publicly available TBI omics datasets (e.g., [139, 140]) have the potential to be used to study cell–cell interaction between T cell subsets and other critical cell populations in this context. A similar approach was recently used to show that CD8+ T cell infiltration of the brain after subarachnoid hemorrhage was dependent on CXCL12 released from vascular endothelial cells and pericytes near the ictus; blockade of this interaction significantly reduces CD8+ T cell infiltration and BBB dysfunction [141]. Applying such methods in TBI and validating identified interactions using complementary molecular techniques including metabolomics and protein‐based assays, amongst others, may elucidate previously unknown interactions between T cell subsets and other cell types like vascular endothelial cells, glia, and neurons in the recovery period after head injury.

It has also not been well established which specific immunogenic cues are responsible for initiating the T cell response, what factors influence their production and release from the CNS, and what resultant mechanisms underlie T cell activation, differentiation, and expansion following head trauma. This is particularly relevant in the context of developing new or repurposing existing therapeutics for application in TBI. The majority of the interventions reviewed herein physically prevent T cells that have already been activated in the periphery from reaching the brain, which successfully blocks effector T cell functioning at the cost of also blocking anti‐inflammatory subsets from being able to dampen the response of other immune cells at the injury site. In contrast, an ideal therapeutic intervention would be subset specific, aiming to selectively prevent the activation of detrimental T cell subsets while bolstering the tolerogenic response of neuroprotective subsets. Considering factors such as the biochemical affinity of antigen to the TCR and the cumulative dose of the TCR‐MHC interaction are known to be involved in shaping the phenotypic differentiation of naïve T cells [142, 143, 144, 145, 146], pharmacologically modulating these initial steps of T cell recruitment post‐injury may be a feasible approach for achieving this. Hence, a deeper understanding of the mechanisms underlying T cell activation and differentiation in TBI is an important area of focus moving forward and may warrant applying single cell RNA sequencing or proteomic techniques to identify how therapeutic treatment alters T cell activation at the signaling level to exert its beneficial effects.

Finally, given that T cells persist in the parenchyma for weeks to months post‐injury [10, 11, 12, 47, 53, 54], characteristics surrounding the longevity of their response may be critical factors to consider when it pertains to therapy development. It remains unknown why T cells do not undergo apoptosis in the time period remote from primary injury like many other responding peripheral immune cells do. Furthermore, it has not been well studied how T cell subset populations within the cortex and bloodstream evolve post‐injury and how this may alter their role in recovery as the majority of the current literature focuses on the T cell response within the acute and subacute periods after injury. It is likely that the T cell landscape undergoes functional transitions at certain timepoints during recovery as it switches from acute pro‐inflammatory functioning toward supporting reparative processes. For instance, in ischemic stroke, pro‐inflammatory T cell subsets including CD8+ and γδ T cells are highly involved in the early pro‐inflammatory response to injury [85, 147]. However, Tregs steadily increase in numbers from 1 to 5 weeks post‐ischemic stroke [148, 149], which supports oligodendrogenesis and white matter repair by promoting tissue‐reparative gene expression in microglia [148]. Similar processes may indeed occur in TBI, and this could underlie the chronic, bimodal increase in T cell numbers within the brain that has been reported in TBI previously. Nevertheless, unraveling this will first require a more granular view of the dynamics of the different T cell subsets' responses to head trauma, which is particularly necessary for T cell populations including Th2 and γδ T cells that are currently understudied in TBI. Analysis using flow cytometry and other cell counting techniques is a logical starting point for achieving this. Understanding the temporal composition of the response is also essential for more finely developing T cell targeting strategies. The reviewed preclinical modulation studies in TBI primarily target acute T cell functioning after primary injury. Special consideration for the temporal trends of specific T cell subsets in the recovery period may identify other critical periods after injury for targeting the activity of individual subsets to better augment the treatment's efficacy and, conversely, may also identify timepoints where pharmacologically altering T cell activity may be harmful.

In summary, while T cells have emerged as key regulators of the neuroimmune response following TBI, a more nuanced understanding of their activation, differentiation, and interactions with other cell populations remains essential. Advancing our knowledge through high‐resolution omics approaches, molecular validation techniques, and longitudinal studies of T cell dynamics will be critical for informing the development of targeted immunomodulatory therapies. Ultimately, tailoring interventions to selectively enhance or suppress specific T cell subsets at appropriate time points post‐injury holds promise for reducing neuroinflammation‐induced secondary injury and ultimately facilitating recovery in TBI patients.

## Author Contributions

**Mitchell D. Kilgore:** conceptualization, methodology, writing – original draft, writing – review and editing, visualization. **Yuwen Xiu:** conceptualization, methodology, writing – review and editing. **Yinghua Jiang:** writing – review and editing. **Yingjie Wang:** writing – review and editing. **Mengxuan Shi:** writing – review and editing. **Di Zhou:** writing – review and editing. **Thin Sein:** writing – review and editing. **Sammy J. Vodovoz:** writing – review and editing, visualization. **Danni Wang:** writing – review and editing. **Aaron S. Dumont:** writing – review and editing, supervision. **Aimee Aysenne:** writing – review and editing, supervision. **Ning Liu:** conceptualization, methodology, writing – review and editing, supervision, funding acquisition. **Xiaoying Wang:** conceptualization, methodology, writing – review and editing, supervision, funding acquisition.

## Conflicts of Interest

Dr. Xiaoying Wang is an Editorial Board member of CNS Neuroscience & Therapeutics and a co‐author of this article. To minimize bias, he was excluded from all editorial decision making related to the acceptance of this article for publication. The remaining authors declare no conflicts of interest.

## Data Availability

Data sharing is not applicable to this article as no new data were created or analyzed in this study.
